# Hexokinase Is Required for Sex Pheromone Biosynthesis in *Helicoverpa armigera*

**DOI:** 10.3390/insects12100889

**Published:** 2021-09-30

**Authors:** Yanpeng Chang, Yunhui Zhang, Zichen Geng, Shuangyan Yao, Wenli Zhao, Xinming Yin, Shiheng An

**Affiliations:** State Key Laboratory of Wheat and Maize Crop Science, College of Plant Protection, Henan Agricultural University, Zhengzhou 450002, China; FSRMchang@163.com (Y.C.); 13403721591@163.com (Y.Z.); 15093972662@163.com (Z.G.); yao8890@yeah.net (S.Y.); xinmingyin@hotmail.com (X.Y.); anshiheng@aliyun.com (S.A.)

**Keywords:** *Helicoverpa armigera*, hexokinase, *Z*11-16:Ald, mating behavior, PBAN/PKA

## Abstract

**Simple Summary:**

Sex pheromone is an essential chemical signal for insect reproduction. Acetyl-CoA, which is generated from glycolysis, is the precursor of sex pheromone biosynthesis in *Helicoverpa armigera*. As the first speed-limited enzyme in glycolysis, the function of Hexokinase (HK) in sex pheromone biosynthesis remains elusive. This article employed *H. armigera* as a model. Results exhibited that the transcription profile of *HaHK* in female moth PGs was consistent with the release trend of sex pheromone. Knockdown of *HaHK* in female PGs caused significant decreases in acetyl-CoA content, sex pheromone production, and mating behaviors. Sugar feeding increased the transcription and enzymatic activity of HK. PBAN signal phospho-activated HaHK in PGs and Sf9 cells via PKA. In general, our study confirmed that PBAN/cAMP/PKA signal activated HaHK activity, and HaHK is required for sex pheromone biosynthesis in *H. armigera*.

**Abstract:**

Acetyl-CoA, the precursor of sex pheromone biosynthesis in *Helicoverpa armigera*, is generated from glycolysis. As the first speed-limited enzyme in glycolysis, Hexokinase (HK) plays an important role in acetyl-CoA production. However, the function of HK in sex pheromone production remains unclear. This study employed *H. armigera* as material to explore the role of HK in sex pheromone production. Results demonstrated that the transcription profile of *HaHK* in female moth pheromone glands (PGs) was consistent with the release fluctuation of sex pheromone. Interference of *HaHK* prevented the increase of acetyl-CoA content induced by PBAN. Therefore, knockdown of *HaHK* in female PGs caused significant decreases in (*Z*)-11-hexadecenal (*Z*11-16:Ald) production, female capability to attract males, and mating rate. Furthermore, sugar feeding (5% sugar) increased the transcription and enzymatic activity of HK. Pheromone biosynthesis activating neuropeptide (PBAN) signal phospho-activated HaHK in PGs and Sf9 cells via protein kinase A (PKA), as shown by pharmacological inhibitor analysis. In general, our study confirmed that PBAN/cAMP/PKA signal activated HaHK, in turn promoted glycolysis to ensure the supply of acetyl-CoA, and finally facilitated sex pheromone biosynthesis and subsequent mating behavior.

## 1. Introduction

Insects are the most prosperous animal group on Earth, partly because of their reproductive capacity. However, successful reproduction relies heavily on female encountering males. In insects, especially lepidopteran moths, females use chemical signals (sex pheromones) to attract males from long-distance for successful mating [1,2]. A sex pheromone is an essential chemical signal that enables the sexes to meet and plays a vital role in insect reproduction [3]. Most female moths produce sex pheromones in a specialized gland called the pheromone gland (PG) through biosynthesis of fatty acids followed by serials of modifications by catalysis with the corresponding enzymes [3]. Pheromone biosynthesis activating neuropeptide (PBAN) is an activator that mediates sex pheromone biosynthesis in most moths [4]. PBAN is produced in the suboesophageal ganglion, released to the hemolymph, arrives at its target tissues the PGs, and binds to the PBAN receptor (PBANR), a G protein-coupled receptor, ultimately initiating sex pheromone biosynthesis in the female PGs [4].

PBAN signaling has been elucidated in *Bombyx mori* and other *Heliothis* species since PBAN was first identified in *Helicoverpa zea* [5]. In *B. mori*, on binding to PBANR, PBAN activates an influx of extracellular Ca^2+^, which activates calcineurin (CaN) and calmodulin-dependent kinase II (CamKII). CaN activates fatty acid reductase, while CamKII promotes the lipolytic release of pheromone precursors by phosphorylating lipid storage droplet protein, and finally facilitates the biosynthesis of sex pheromones [6]. In *Helicoverpa. armigera*, PBAN uses Ca^2+^ and cAMP as second messengers when it binds with PBANR. Ca^2+^ activates CaM to form the CaM/CaN complex, and then activates acetyl CoA-carboxylase (ACC: the rate-limiting enzyme of fatty acid biosynthesis) by dephosphorylation. The cAMP/PKA signal indirectly activates ACC by inhibiting adenosine 5′-monophosphate (AMP)-activated protein kinase (AMPK), finally ensuring sex pheromone biosynthesis [7].

Acetyl-CoA is the precursor of sex pheromone biosynthesis in most moths [1]. Sex pheromones are biosynthesized from acetyl-CoA via fatty acid synthesis and chain modification. Acetyl-CoA generates from glucose (mainly), fatty acids, and amino acid catabolism [8,9]. In insects, trehalose is immediately transported to various tissues through the hemolymph after being biosynthesized in the fat body [10]. Then trehalose is hydrolyzed to glucose by trehalase 1 in the cytoplasm [11] and trehalase 2 in the cell membrane [12]. As the most important source of cellular energy, glucose enters glycolysis under anaerobic conditions to generate pyruvate. Then, pyruvate undergoes oxidative decarboxylation to generate acetyl-CoA, which enters the tricarboxylic acid cycle and undergoes oxidative phosphorylation, releasing large amounts of ATP [13]. Glycolysis precedes the aerobic oxidation of sugars to generate acetyl-CoA. Furthermore, hemolymph trehalose is used directly in sex pheromone biosynthesis. For example, in *Heliothis virescens*, most sex pheromone production follows a single adult feeding, which is used to generate trehalose and acetyl-CoA [14,15]. Similarly, in *Mythimna separata*, sugar feeding promotes to sex pheromone biosynthesis by increasing trehalose, pyruvic acid, and acyl-CoA levels in the PGs [16]. In addition, in *H. armigera*, 5% sugar feeding significantly increases the production of (*Z*)-11-hexadecenal (*Z*11-16:Ald, the main components of sex pheromones) [17]. These results explain the importance of feeding on sex pheromones. Acyl-CoA is rapidly used to biosynthesize sex pheromones.

Since acetyl-CoA is generated mainly from pyruvic acid in glycolysis and acts as the precursor of sex pheromone biosynthesis, glycolysis is crucial for sex pheromone biosynthesis. Three rate-limiting enzymes regulate glycolysis: hexokinase (HK), 6-phosphofructokinase1 (PFK1), and pyruvate kinase (PYK) [18,19,20]. HK is the first key enzyme by which cells control the rate of glucose metabolism in glycolysis [21,22]. Mammals have four HK isoenzymes: HK1 to HK4. These have different tissue and subcellular distributions, developmental stage characteristics, and enzyme kinetics [23]. In insects, *Tribolium castaneum TcHKA1* RNAi causes embryonic lethality and fewer eggs to be laid [24]. In *Nilaparvata lugens* fecundity, *HK*-*1* functions by affecting glycometabolism, protein synthesis, and vitellogenin expression [25]. *H. armigera* hexokinase (HaHK, Genbank NO. KR780750.1) expression and activity levels are decreased in diapause-destined pupae; interference with *HaHK* reduces metabolic activity, increases ROS activity, and ultimately delays pupal development [26]. However, the function and mechanism of HK in sex pheromone biosynthesis remains unclear. This study explored the function of HaHK in *H. armigera* in sex pheromone production and mating behavior after knockdown of *HaHK*, and investigated the functional mechanism of HaHK by measuring enzymatic activities and phosphorylation levels on the condition of PBAN treatment.

## 2. Materials and Methods

### 2.1. Insects

*H. armigera* larvae and moths were fed with artificial diet in laboratory with 26 ± 1 °C temperature, 75 ± 1% humidity, and14L:10D photoperiod [27]. The feed recipes for larvae and moths were listed in the Table S1 and Table S2, respectively.

### 2.2. Cell Culture

Sf9 cells were cultured with Sf-900^TM^ II SFM (containing 10% FBS, 0.5% Penicillin-Streptomycin Liquid) at 28 °C. FBS and Penicillin–Streptomycin Liquid were from the Gibco Company (Gibco, Carlsbad, CA, USA). 

### 2.3. Chemicals

The active PBAN fragment (Ser-Arg-Thr-Lys-Tyr-Phe-Ser-Pro-Arg-Leu-NH_2_) was synthesized in the Sangon Company (Sangon, Shanghai, China). *Z*11-16:Ald was from the Sigma Company (Sigma, St. Louis, MO, USA) and used to quantify sex pheromone content by gas chromatography (GC) referred to the previous study [2].

### 2.4. qRT-PCR

Total RNA from the fat body, epidermis, muscle, and PG were extracted by RNA-easy Isolation Reagent according to the manufacturer’s instructions (Vazyme, Nanjing, China). The cDNA was synthesized by the HiScript III RT SuperMix kit (+gDNA cursor) (Vazyme, Nanjing, China) and then used as the template of qRT-PCR. The internal reference gene was *18s* (*18S*-RTF: GCATCTTTCAAATGTCTGC; *18S*-RTR: TACTCATTCCGATTACGAG) [28]. The qRT-PCR analysis of *HaHK* (*HaHK*-RTF: AGCGGTGGACCAAAGGATT; *HaHK*-RTR: TCCAGTGCCAACGATGAGT) was performed by ChamQ Universal SYBR qPCR Master Mix (Vazyme, Nanjing, China) and Applied Biosysterms 7500 fast real-time PCR instrument (ABI, Carlsbad, CA, USA). The qPCR procedures were set as following, 95 °C for 5 min, followed by 40 cycles of 95 °C for 15 s and 60 °C for 20 s. The comparative Cross Threshold method was used to quantify the relative expression level [29]. Three biological replicates and three technical repetitions were employed. The significant differences were compared by Student’s *t*-test or Tukey test.

### 2.5. dsRNA Synthesis

The DNA template (686 bp) for ds*HaHK* synthesis was amplified by PCR with specific primers containing T7 promoter sequence (*HaHK*-T7F: GATCACTAATACGACTCACTATAGGGAGAGACGCCATTGCCAGACGAGG; *HaHK*-T7R: GATCACTAATACGACTCACTATAGGGAGAGTGGGTGGAAACGGTAGACG). The enhanced green fluorescent protein (EGFP, GenBank NO. MN623123.1, 422 bp portion of the EGFP sequence) ds*RNA* was synthesized as the negative control (*EGFP*-T7F: GATCACTAATACGACTCACTATAGGGAGACACAAGTTCAGCGTGTCCG; *EGFP*-T7R: GATCACTAATACGACTCACTATAGGGAGAGTTCACCTTGATGCCGTTC). The ds*RNA* was synthesized by T7 RNAi Transcription kit according to the manufacturer’s instructions in vitro (Vazyme, Nanjing, China). The ds*RNA* quality and concentration were measured by a biophotometer (Eppendorf) and agarose gel electrophoresis. 

### 2.6. Measurement of Z11-16:Ald Content

The newly-emerged female moths were decapitated in order to remove endogenous PBAN. The decapitated-females were then injected with ds*HaHK* (10 μg), after 48 h, the females were injected with PBAN (10 pM). One hour later, PGs were collected (15 PGs in one group) and dissolved into hexane and subjected to sex pheromone measurement by GC method according to previous reference [2]. Briefly, the sex pheromone component (*Z*11-16: Ald) was measured by a GC instrument (Trace GC Ultra Trace DSQ; MS-Thermo Scientific DSQ II) equipped with a 30 m capillary column (RTX-5SILMS, Restek, 0.25 mm diameter). The programs were listed as following: the temperature was maintained at 150 °C for 5 min; then increased to 230 °C at 30 °C/min and maintained 5 min, during this period all sex pheromone components were eluted. Finally, the column was heated to 260 °C at 20 °C/min and held at this temperature for 3 min (the last sample addition time was 15 min for the cleaning of column). *Z*11-16:Ald were identified and measured by the retention time with standard sample of *Z*11-16:Ald and corresponding area of *Z*11-16:Ald peak, respectively. The DEPC+PBAN-injected (3 μL+ 10 pM, respectively) females and ds*EGFP*+PBAN-injected (10 μg [3 μL] + 10 pM, respectively) females were used as the control groups.

### 2.7. Mating Behavior

The newly-emerged female moths (*n* = 15) were injected with ds*HaHK* (10 μg) and then put into cage (60 × 60 × 60 cm). Twenty-four hours after injection, normal male moths (*n* = 15) were also put into the same cage. After 24 h, mating proportion of females was calculated according to the number of spermatophore-containing females following previous method [28]. The ds*EGFP*-injected (10 μg) females (*n* = 15) and normal male moths (*n* = 15) were put into another cage and employed as negative control. Three biological replicates were employed. 

### 2.8. Female Ability to Attract Male

The newly-emerged females (*n* = 15) were injected with 10 μg ds*HaHK*, after 48 h, the treated-females were placed into the trap cell (28 cm high × 30 cm wide × 30 cm long). The ds*EGFP*-injected females (10 ug, control group) (*n* = 15) were put into another trap cell. Two-day-old normal males (*n* = 50) were placed into the release cell (height 32 cm × width 30 cm × length 60 cm) which located above of the trap cell. After 24 h waiting time, the number of males were calculated. Three biological replicates were performed. The attraction box is shown in Figure S1.

### 2.9. Measurement of Acetyl-CoA Content

PBAN treatment:

The newly-emerged females were decapitated. After 24 h, the isolated PGs of the females were incubated with PBAN (10 pM) for 30 min. The control group were treated with water for 30 min. There were 30 PGs per group.

Knockdown of *HaHK*:

The newly-emerged females were decapitated, 24 h later, the control group 1 was injected with DEPC (the solvent of dsRNA, 3 μL), the control group 2 was injected with ds*EGFP* (10 μg, 3 μL), and the experimental group was injected with ds*HaHK* (10 μg, 3 μL). After waiting 24 h, the PGs were isolated and incubated with PBAN (10 pM) for 30 min. There were 30 PGs per group.

Then above-treated PGs were subjected to the measurement of acetyl-CoA content according to the instrument book of the Acetyl-CoA content determination kit (G0826W) (Grace Biotechnology Company, Suzhou, China).

### 2.10. Sugar Feeding

Newly-emerged females (*n* = 20) were reared in a cage (30 × 30 × 30 cm) with 5% sugar and PGs were collected at different time points (24, 48 and 72 h) for qRT-PCR and enzymatic activity analyses [30]. Water-fed females were used as the control.

### 2.11. Subcellular Localization

HaHK CDS sequence was ligated into RFP-His-pIEx-4 vector to construct HaHK-RFP-His-pIEx-4 plasmid with specific primers (HaHK-SacI-RFP-F: ATCGTTAACACGTCAAGAGCTCATGCGTCAAGCACGAGAT, HaHK-BglII-RFP-R: TGCAGGCGCGCCGAGATCTGCGCGACAGTACTGCTTTG). HaHK-RFP-His-pIEx-4 was transfected into Sf9 cells using FuGENE^®^ HD Transfection Reagent as previously described (Promega, Madison, WI, USA, E2311) [31]. Cells transfected with RFP-His-pIEx-4 plasmid were used as the control. The fluorescence images were photographed by LSM710 laser confocal (Zeiss, Oberkohen, Germany).

### 2.12. Co-Immunoprecipitation (Co-IP)

The effect of PBAN treatment on phosphorylation levels of HaHK:

The Sf9 cells (about 3.6 × 10^6^ cells in one well of the 6 cell culture cluster, use 3.6 × 10^6^ cells for one Co-IP experiment) were transfected with HaHK-RFP-His-pIEx-4 (5 μg plasmid) using FuGENE^®^ HD Transfection Reagent as previously described [31]. After 48 h waiting time, cells were incubated with PBAN (10 pM). Treated cells were harvested at different time after PBAN treatment (0, 10, 30, 60 and 90 min) and then dissolved by lysis buffer RIPA (300 μL). The protein concentration was determined by the BCA protein assay kit (Solarbio, Beijing, China). Then some lysate solution (60 μL) was taken as the input. Anti-phosphoserine antibody (Abcam, ab9332) was added to the residual lysate and the samples were rotated at 4 °C for 8 h as previously described [32]. Then Pierce™ Protein A/G Magnetic beads (ThermoFisher^TM^, 88802) were mixed with samples for 2 h. Finally, the immunoprecipitation complex was subjected to Western blot. 

The effect of PBAN and H-89 treatments on phosphorylation levels of HaHK:

The Sf9 cells (about 3.6 × 10^6^ cells in one well of the 6 cell cultrue cluster, use 3.6 × 10^6^ cells for one Co-IP experiment) were transfected with HaHK-RFP-His-pIEx-4 (5 μg plasmid) using FuGENE^®^ HD Transfection Reagent as previously described [31]. After 48 h waiting time, cells were incubated with PKA inhibitor H-89 (20 μM) for 1 h followed by PBAN (10 pM) treatment for different time points (0, 10, 30, 60 and 90 min). Treated cells were harvested at different time after PBAN treatment (0, 10, 30, 60 and 90 min) and then dissolved by lysis buffer RIPA (300 μL). The protein concentration was determined by the BCA protein assay kit (Solarbio, Beijing, China). Then some lysate (60 μL) was taken as the input. Anti-phosphoserine antibody (Abcam, ab9332) was added to the residual lysate and rotated at 4 °C for 8 h, as described [32]. Pierce™ Protein A/G Magnetic beads (ThermoFisher^TM^, 88802) were mixed with samples for 2 h. Finally, the immunoprecipitation complexes were subjected to Western blot.

### 2.13. Western Blot

Treated samples were denatured and separated by SDS-polyacrylamide gel electrophoresis (Zhonghuihecai, Xian, China, PE008) and then transferred to PVDF membrane (Roche, Basel, Switzerland, 3010040001). The membrane was incubated with TBST containing 5% skimmed milk for 1 h at room temperature and reacted with the His monoclonal antibody (Abbkine, Wuhan, China, A02050) for 1 h. The membrane was incubated HRP-labeled secondary antibody. The signal was detected using superlumia ECL plus HRP substrate kit (Abbkine, Wuhan, China, K22030) according to manufacturer’s instruction.

### 2.14. HaHK Enzymatic Activity Measurement

HaHK enzymatic activity in isolated PGs:

PGs from 2-day-old females (2 days after emergence) were dissected and then incubated with Graces’ medium for 2 h. Then above medium was replaced with new Graces’ medium containing 10 pM PBAN. PGs were collected at different time after PBAN treatments (0, 10, 30, 60 and 90 min) and subjected to HaHK activity analysis by HK activity kit (Solarbio, BC0745) according to the manufacturer’s instruction [16]. Thirty PGs per time point.

PGs were collected from 2-day-old females and then incubated with Graces’ medium for 2 h. Above medium was replaced with new medium containing 20 μM H-89 (PKA inhibitor) or 10 μM CC (chelerythrine chloride, PKC inhibitor) for 1 h. Then the medium was added with PBAN (10 pM). PGs were collected at different time points after PBAN treatments (0, 10, 30, 60 and 90 min) and subjected to HaHK activity measurement by HK activity kit (Solarbio, BC0745). There were 30 PGs per time point. Three biological replicates were performed.

HaHK enzymatic activity in Sf9 cells:

The Sf9 cells were transfected with HaHK-RFP-His-pIEx-4 plasmid using FuGENE^®^ HD Transfection Reagent as previously described [31]. After 48 h waiting time, cells were incubated with PBAN (10 pM) (PBAN activates the endogenous PBANR expression of Sf9 cells) at different time points (0, 10, 30, 60 and 90 min) and then subjected to HaHK activity measurement using HK activity kit (Solarbio, Beijing, China, BC0745) following to the manufacturer’s instruction. Three biological replicates were employed.

## 3. Results

### 3.1. Expression Profile of HaHK in the PGs

**Figure 1:**
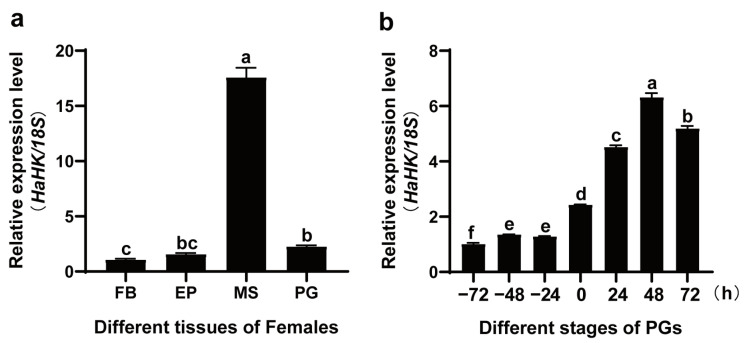
The expression pattern of *HaHK* by qRT-PCR. (**a**) The tissue distribution of *HaHK* in female moths. FB: fat body, EP: epidermis, MS: muscle, PG: pheromone gland. (**b**) The transcription of *HaHK* in the PG at different developmental stages. 0 h: The emergence time. The internal reference gene was *18S*. Error bars were the mean ± s.d. of three independent biological experiments. Different letters indicated significant differences at *p* < 0.05 level by Tukey test.

Using qRT-PCR, *HaHK* was distributed in the fat body, epidermis, flight muscle, and PGs (Figure 1a). In the PGs, the transcription of *HaHK* was detected at 72 h before emergence, and increase after emergence, peaking at 48 h after emergence (Figure 1b).

### 3.2. Knockdown of HaHK Decreased Acetyl-CoA Content, Sex Pheromone Production, and Mating Behaviors

**Figure 2:**
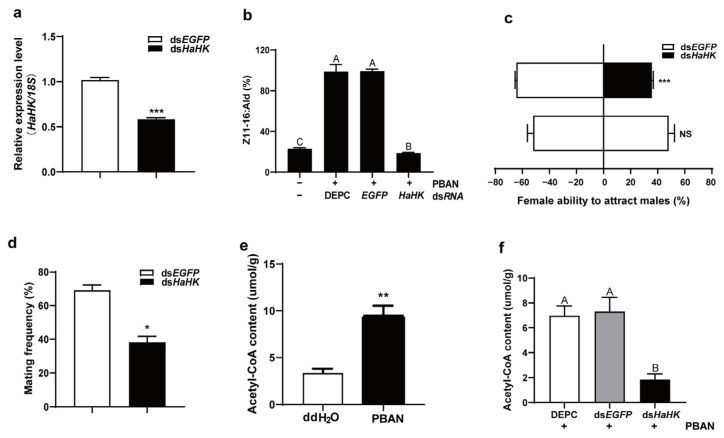
Interference of *HaHK* decreased the contents of acetyl-CoA and sex pheromone and mating. (**a**,**b**) The sex pheromone contents in female PGs (**b**) after knockdown of *HaHK* (**a**). Females were injected with DEPC (3 μL), ds*HaHK* (10 μg, 3 μL), or ds*EGFP* (10 μg, 3 μL). Error bars were the mean ± s.d. of three independent biological experiments. *** *p* < 0.001, Student’s *t*-test. Different letters indicated significant differences at *p* < 0.01 level by Tukey test. (**c**,**d**) The female ability to attract males (**c**) and mating frequency (**d**) after knockdown of *HaHK* by RNAi. ds*HaHK* (10 μg), ds*EGFP* (10 μg, the control). Error bars were the mean ± s.d. of three independent biological experiments, * *p* < 0.05, *** *p* < 0.001, Student’s *t*-test. (**e**) The content of acetyl-CoA in the isolated PGs after PBAN treatment. Thirty PGs were treated with PBAN (10 pM, 30 min). The control was incubated with ddH_2_O. Error bars were the mean ± s.d. of three independent biological experiments. ** *p* < 0.01, Student’s *t*-test. (**f**) The content of acetyl-CoA after knockdown of *HaHK*. Females were treated with DEPC (3 μL), ds*EGFP* (10 μg, 3 μL), or ds*HaHK* (10 μg, 3 μL). PBAN (10 pM, 30 min). There were 30 PGs in each group. Error bars were the mean ± s.d. of three independent biological experiments. Different letters indicated significant differences at *p* < 0.01 level by Tukey test.

**Figure 3:**
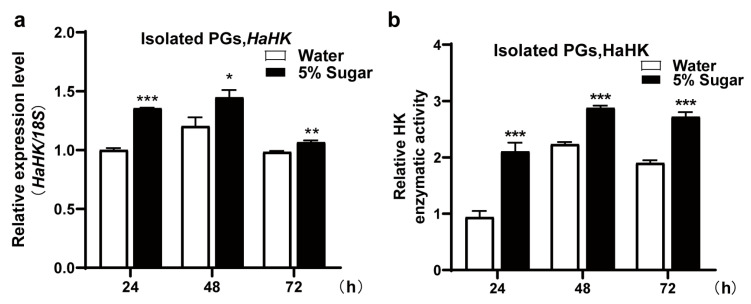
Sugar feeding increased the transcription and enzymatic activity of HaHK. (**a**) The transcription changes of *HaHK* in PGs under 5% sugar or water feeding for different time (24, 48 and 72 h) by qRT-PCR. The internal reference gene was *18S*. Error bars were the mean ± s.d. of three independent biological experiments. * *p* < 0.05, ** *p* < 0.01, *** *p* < 0.001, Student’s *t*-test. (**b**) The enzymatic activity of HaHK under 5% sugar feeding for different time (24, 48 and 72 h). Water-fed females were the control group. Error bars indicated the mean ± s.d. of three independent biological experiments. * *p* < 0.05, ** *p* < 0.01, *** *p* < 0.001, Student’s *t*-test.

The injection of ds*HaHK* significantly decreased *HaHK* transcription (Figure 2a). *HaHK* knockdown resulted in a significant decrease in sex pheromone production, compared with the ds*EGFP*-injected control (Figure 2b). Accordingly, the female ability to attract males and mating frequency also decreased in ds*HaHK*-injected females compared with the ds*EGFP*-injected controls (Figure 2c,d). The content of acetyl-CoA was increased under PBAN treatment (Figure 3e). However, knockdown of *HaHK* prevented the increase of acetyl-CoA induced by PBAN, by contrasted with the DEPC+PBAN and ds*EGFP*+PBAN control groups (Figure 3f). These results indicated that *HaHK* is required for sex pheromone biosynthesis and mating behaviors for its necessary role in acetyl-CoA production.

### 3.3. Sugar Feeding Increased the Transcription and Enzymatic Activity of HaHK

Feeding with 5% sugar significantly up-regulated *HaHK* transcription at 24, 48, and 72 h, compared with control females fed water (Figure 3a). The HaHK enzymatic activity was also significantly increased at 24, 48, and 72 h after sugar feeding (Figure 3b), implying that HaHK responds to sugar feeding with increased transcription and enzymatic activity.

### 3.4. PBAN Activated Enzymatic Activity of HaHK via PKA

**Figure 4:**
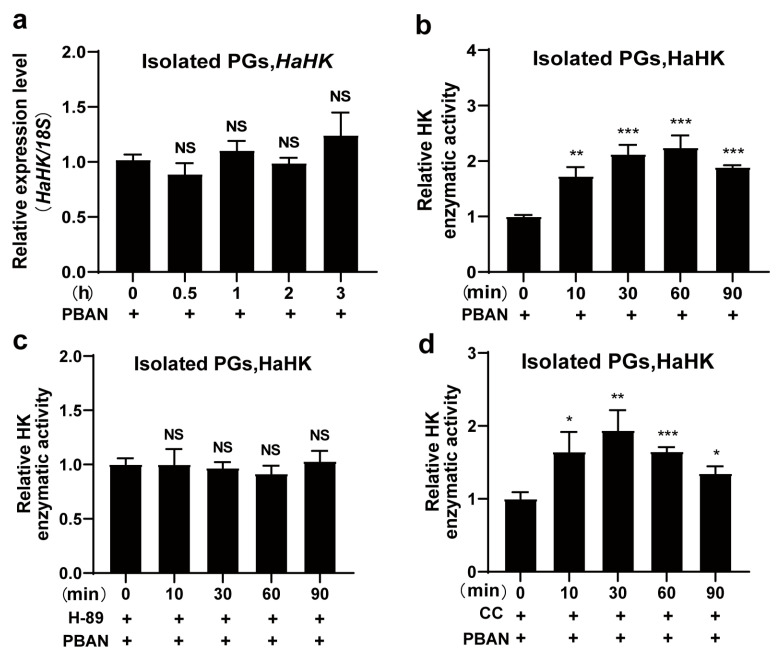
PBAN activated HaHK activity via PKA. (**a**) The transcription of *HaHK* under PBAN treatment for different time points (0, 0.5, 1,2, and 3 h). *18S* was the internal reference. The isolated PGs were incubated with PBAN (10 pM). Error bars indicated the mean ± s.d. of three independent biological experiments. (**b**) The enzymatic activities of HaHK under PBAN treatment in isolated PGs. PBAN (10 pM), time points: 0, 10, 30, 60, and 90 min. ** *p* < 0.01, *** *p* < 0.001, Student’s *t*-test. (**c**,**d**) The enzymatic activity of HaHK under H-89 (PKA inhibitor) + PBAN (**c**) or CC(PKC inhibitor) + PBAN (**d**) treatments. PBAN (10 pM), H-89 (20 μM, 1 h), CC (10 μM, 1 h), time points: 0, 10, 30, 60, and 90 min. Error bars were the mean ± s.d. of three independent biological experiments, * *p* < 0.05, ** *p* < 0.01, *** *p* < 0.001, Student’s *t*-test.

Using qRT-PCR, PBAN treatment had no effect on the expression of *HaHK* mRNA (Figure 4a). Notably, PBAN treatment significantly increased the enzymatic activity of HaHK in isolated PGs (Figure 4b), and H-89 (a specific inhibitor of PKA) prevented the enzymatic activity of HaHK induced by PBAN (Figure 4c). However, CC (a PKC inhibitor) treatment did not attenuate the PBAN-induced increase in the enzymatic activity of HaHK (Figure 4d). These results imply that PBAN activates HaHK activity via PKA signaling.

### 3.5. PBAN Induced the Phosphorylation and Activation of HaHK via PKA

**Figure 5:**
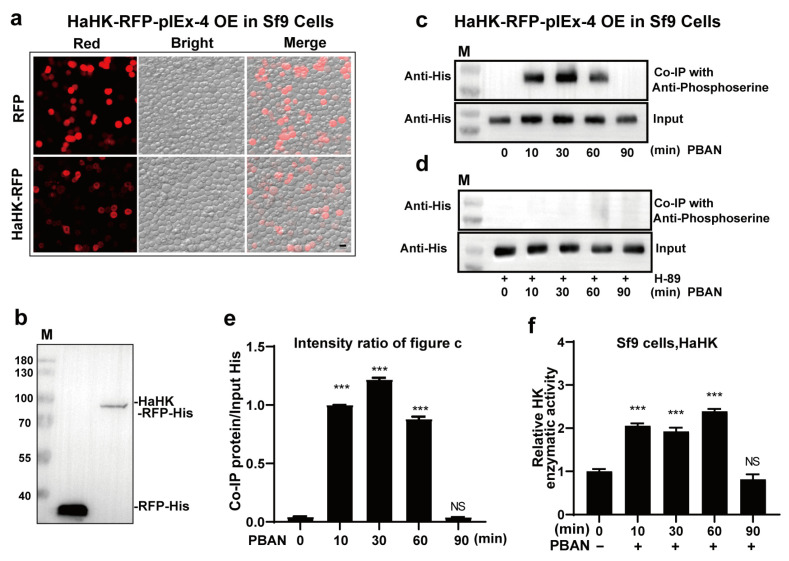
PBAN induced PKA to phosphorylate and activate HaHK activity. (**a**) The subcellular localization of HaHK-RFP-His in Sf9 cells. Cells transfected with RFP-His were used as the control. Merger was the overlap of the red and bright. Bar was 10 μm. (**b**) The expression of HaHK-RFP-His and RFP-His proteins were verified by WB by His antibody. M: protein marker. (**c**,**d**) The phosphorylation levels of HaHK-RFP-His under PBAN (**c**) or H-89+PBAN (**d**) treatments in Sf9 cells by Co-IP. PBAN (10 pM), H-89 (20 μM, 1 h). (**e**) Statistical analysis of data in c by Image J. Error bars indicated the mean ± s.d. of three independent biological experiments, *** *p* < 0.001, Student’s *t*-test.(**f**) The enzymatic activity of HaHK-RFP-His under PBAN treatment in Sf9 cells. PBAN (10 pM). Error bars indicated the mean ± s.d. of three independent biological experiments, *** *p* < 0.001, Student’s *t*-test.

HaHK-RFP-His-pIEx-4 was successfully expressed in Sf9 cells and was located in the cytoplasm based on fluorescent images and Western blotting (Figure 5a,b). PBAN treatment led to a significant increase in the phosphorylation level of HaHK, as shown by Co-IP (Figure 5c,e and Figure S3). However, the increased phosphorylation of HaHK induced by PBAN was significantly attenuated by H-89 (Figure 5d and Figure S3), indicating that PKA induced the phosphorylation of HaHK. Correspondingly, the HaHK-RFP-His activity significantly increased in response to PBAN treatment (Figure 5f). These results imply that PBAN recruits PKA to phosphorylate and activate HaHK.

## 4. Discussion

The primary precursor for the de novo biosynthesis of most sex pheromone is acetyl-CoA [1,6]. In glycolysis, glucose generated pyruvates, which is decarboxylated to form acetyl-CoA. Importantly, studies in *H. virescens*, *M. separata*, and *H. armigera* proved that sugar feeding increase sex pheromone production in female moths by increasing acetyl-CoA [15,16,17]. These findings imply the importance of supplementary nutrition and subsequent glycolysis in sex pheromone biosynthesis. Therefore, this study used *H. armigera* as a model to investigate the function of HK, a rate-limiting enzyme in glycolysis, in sex pheromone biosynthesis. Besides, the phylogency tree of HK demonstrated that HaHK in present study belongs to HK2 isoform (Figure S2). In order to consistent with the previous study, HaHK is still used in this study. 

HK is the first enzyme in aerobic glycolysis and phosphorylates glucose to generate glucose-6-phosphate [22]. As an important rate-limiting enzyme in the glycolysis pathway, HK plays important roles in many physiological processes. For instance, *CmHK* in *Cnaphalocrocis medinalis* is highly expressed in the ovary and fifth instar larvae, ds*CmHK* injection causes increased larval and pupal mortality and significant variation in the sex ratio; it also significantly reduces ovulation and the larval hatching rate [33]. Sex pheromones are biosynthesized de novo in the PGs from acetyl-CoA, which originates from pyruvate during glycolysis. In present study, data proved that HaHK was necessary for the PBAN-induced increase of acetyl-CoA content (Figure 2). This explains the requirement of HK for sex pheromone biosynthesis and the ability of females to attract males, as demonstrated by the RNAi-mediated knockdown of HK (Figure 2).

Previous studies have shown that HK transcription is regulated. For example, in prostate cancer cells, androgen increases glucose utilization via activation of HK2. Androgen activates cAMP-dependent protein kinase via phosphorylation of the cAMP-response element, which in turn binds to CRE on the HK2 promoter, finally leading to transcriptional activation of HK2 [34]. In adipocytes, HK2 expression is induced by the FRAP/mTOR-dependent pathway and MAPK pathway in response to insulin [35]. In this study, we proved that sugar feeding up-regulated the transcription and enzymatic activity of *HaHK* (Figure 3). Our results are consistent with findings that insulin regulates the transcription of *HK* following sugar feeding, although further studies need to address the mechanism. Most importantly, studies have revealed that HK activity is mediated by phosphorylation. For example, c-Src, the first proto-oncogene identified in animal cells, interacts with and phosphorylates HK1 and HK2. Phosphorylation significantly increases HK enzymatic activity, thereby enhancing glycolysis [36]. Proviral insertion in murine lymphomas 2 kinase directly binds to HK2 and phosphorylates HK2 at Thr^473^, thereby increasing HK2 enzymatic activity [37]. In *Xenopus laevis*, HK is phosphorylated under dehydration and then facilitates glycolytic metabolism in a hypoxic state [38]. Even in yeast, the phosphorylation regulation of HK2 is controlled by Snf1 at Ser^14^ in vivo and in vitro [39]. These findings indicate that HK is phospho-activated. Our study confirmed that the PBAN signal regulates the phosphorylation of HK, thereby promoting the HK enzymatic activity (Figure 5). For the first time, we provide evidence that the insect neuropeptide PBAN regulates HK phosphorylation in the *H. armigera* PGs. The specific phosphorylated sites of HaHK with PBAN treatment need further study.

**Figure 6:**
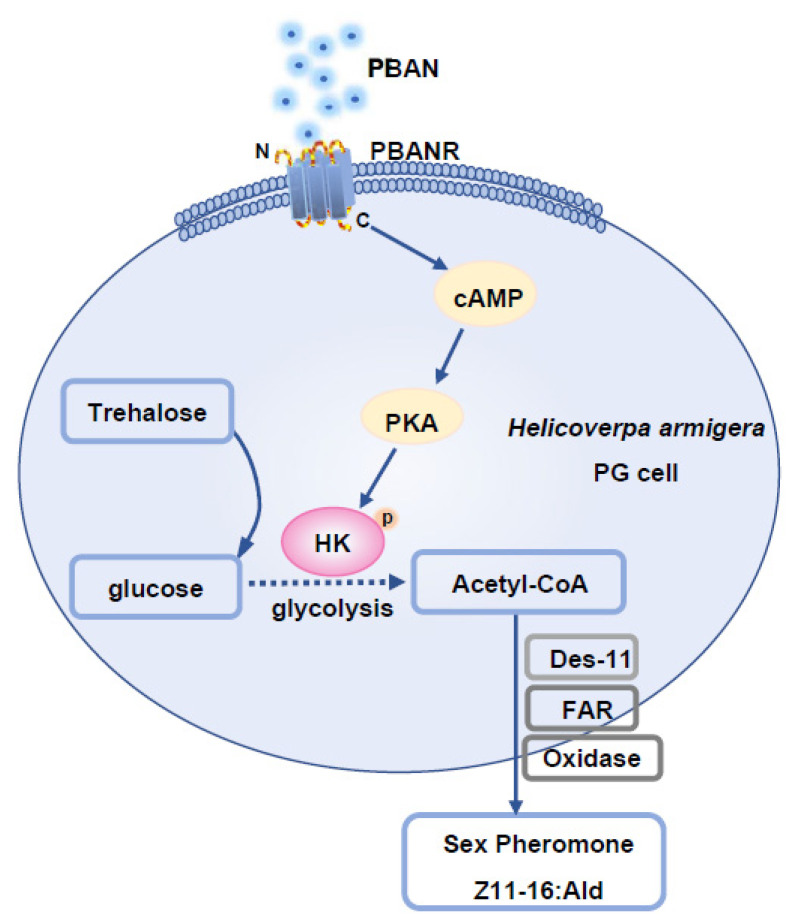
The diagram for *H. armigera* HaHK function in PBAN-induced sex pheromone biosynthesis. PBAN binds to PBANR and recruits cAMP to activate PKA. Then activated-PKA phospho-activates HK. As the rate-limiting enzyme of glycosis, HK controls the production of acetyl-CoA, thus affecting the content of Z11-16:Ald.

PBAN regulates sex pheromone biosynthesis in moths. In *B. mori* [40], *Spodopdera. litura* [41], and *Ostrinis nubilalis* [42], the pheromonotropic effects of PBAN require extracellular Ca^2+^, indicating that PBAN uses Ca^2+^ as a secondary messenger in these species. However, cAMP failed to elevate the response to PBAN binding in isolated PGs of these species [43], indicating that cAMP is not used as a secondary messenger in PBAN signal transduction. Conversely, in heliothine moths, Ca^2+^ and cAMP are required for the pheromonotropic effects of PBAN [44,45,46]. In the species that use Ca^2+^ and cAMP as second messengers, the role of Ca^2+^ has been well elucidated [7]. However, the function of the cAMP/PKA signal remains elusive, although cAMP/PKA signal has been confirmed to play an important role in sex pheromone biosynthesis [44,45,46,47]. Our findings provide important evidence that PBAN activates HK by phosphorylation via PKA, promoting glycolysis, ultimately facilitating sex pheromone biosynthesis and subsequent mating (Figure 2, Figure 4 and Figure 5). PKA-mediated phosphorylation of HK has been reported. For example, in the rat brain, the cAMP/PKA system regulates short-term HK activity during neuronal activity [48]. In yeast, HKI or HK2 mutants impair cAMP production and PKA pathway activation [49]. These findings show the universal regulatory effects of HK. We demonstrated that PBAN not only regulates steps in sex pheromone biosynthesis, but also precursor production. For the first time, we confirmed that PBAN uses PKA to activate HK, ensuring the progress of glycolysis and the sex pheromone precursor biosynthesis, ultimately facilitating sex pheromone biosynthesis (Figure 6).

## Data Availability

Not applicable.

## Supplementary Materials

The following are available online at www.mdpi.com/2075-4450/12/10/889/s1, Figure S1. A diagram of the attraction box. Figure S2. The phylogentic tree analysis of 29 HK proteins. This image was generated from MEGA. Table S1. Feed for *Helicoverpa armigera* larvae. Table S2. Moth feed of *Helicoverpa armigera*.
