# Supplementary material for: Hexokinase Is Required for Sex Pheromone Biosynthesis in Helicoverpa armigera
Source: Insects. 2021 Sep 30;12(10):889. doi: 10.3390/insects12100889 (PMC8541617; doi:10.3390/insects12100889)
Supplement: Supplementary file 1 [file insects-12-00889-s001.zip › insects-1360040-supplementary.pdf]

Hexokinase is required for sex pheromone biosynthesis in *Helicoverpa armigera*

Yanpeng Chang, Yunhui Zhang, Zichen Geng, Shuangyan Yao, Wenli Zhao*, Xinming Yin and Shiheng An

State key Laboratory of Wheat and Maize Crop Science/College of Plant Protection, Henan Agricultural University, Zhengzhou, 450002, China.

***Correspondence:**

Wenli Zhao, E-mail: zhaowenli19900218@163.com

This supplementary contains Figure S1 and Figure S2, table S1 and table S2.

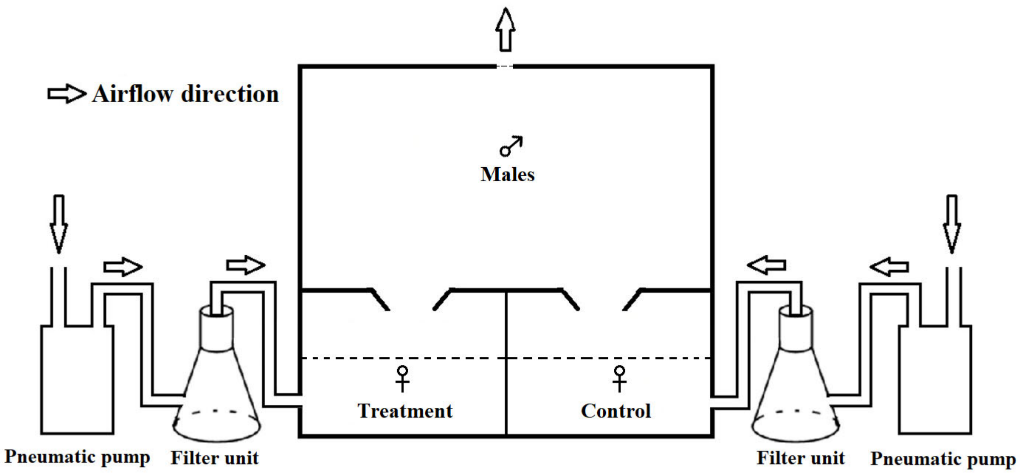

Figure S1. A diagram of the attraction box.

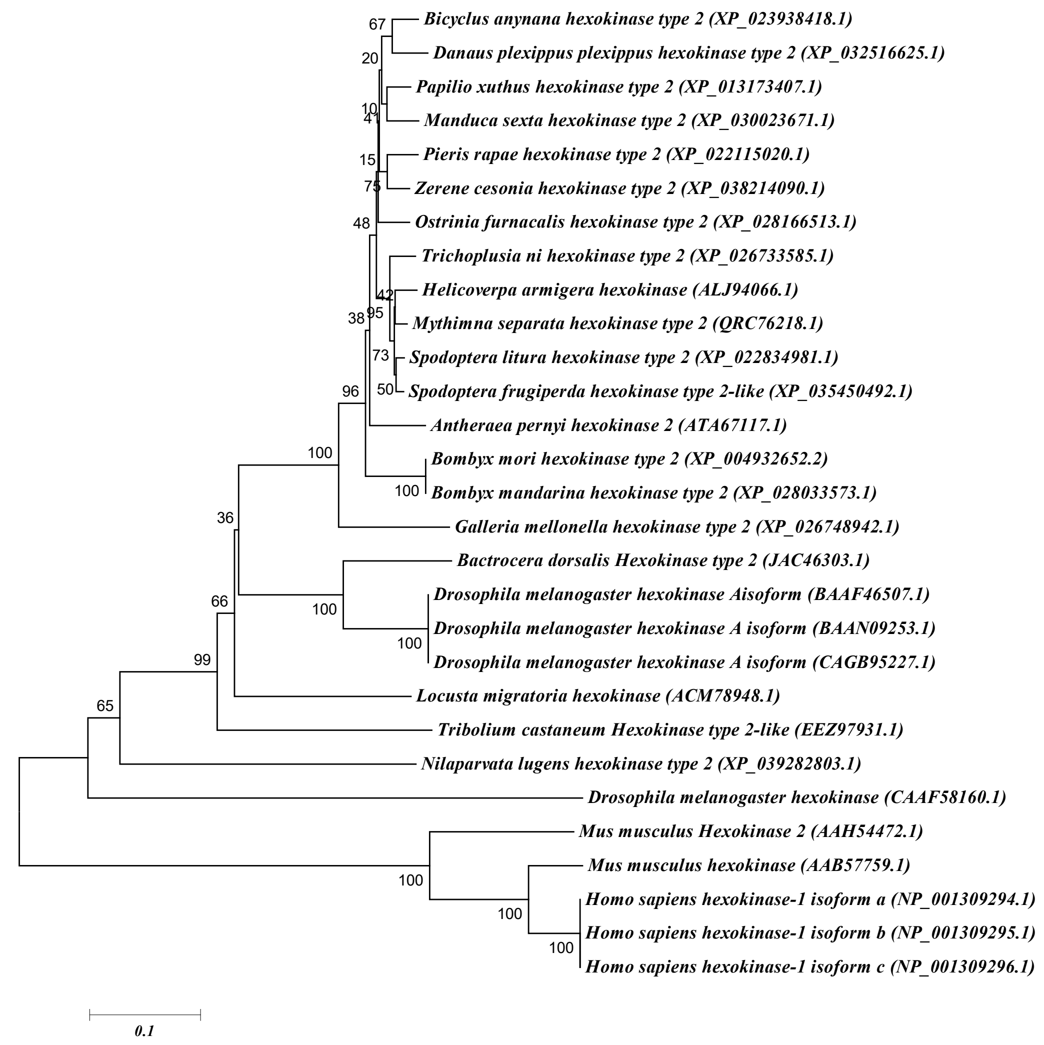

Figure S2. The phylogentic tree analysis of 29 HK proteins. This image was generated from MEGA-X.

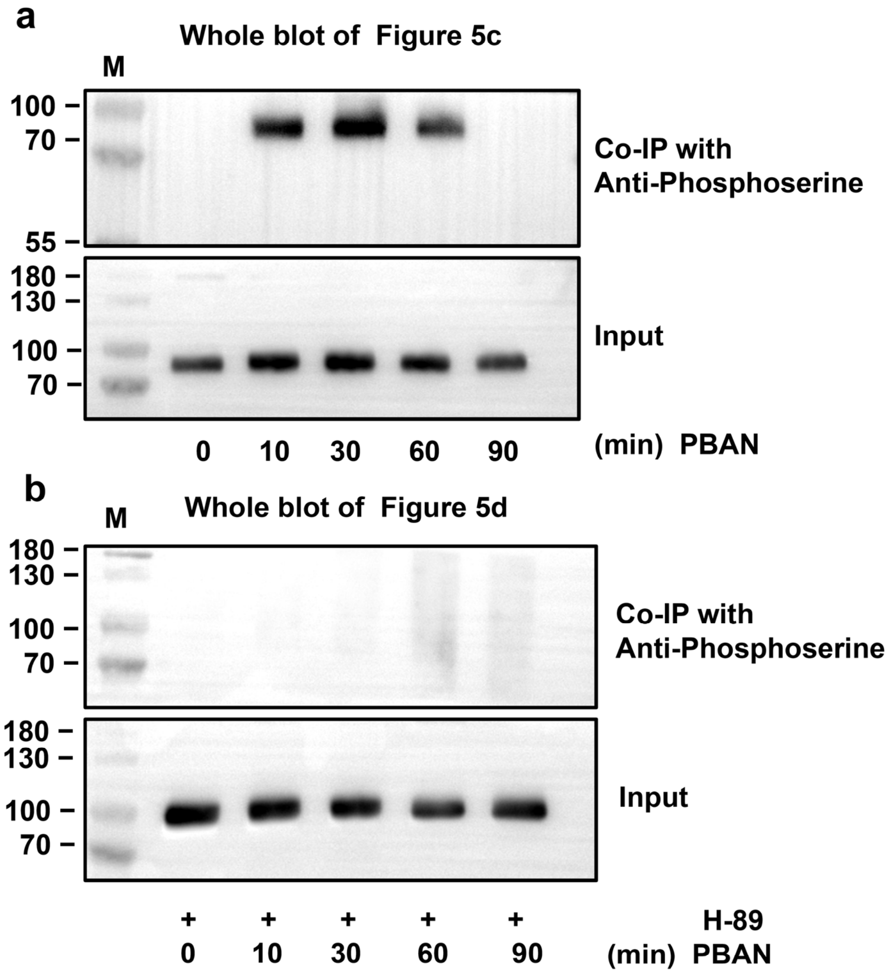

Figure S3. The whole blots of Figure 5c and Figure 5d.

Table S1. Feed for *Helicoverpa armigera* larvae

	Ingredient	amount	Treatment
Compound 1	Soybean flour	160 g	Mixing
	Wheat germ powder	300 g	
	Yeast powder	60 g	
	Sucrose	40 g	
Compound 2	Sorbic acid	6 g	Boiled and then add to compound 1
	Methyl paraben	6 g	
	Water	1200 mL	
	Agar powder	40 g	

Compound 3	Casein	80 g	Boiled and then add to above mixture
	Water	1600 mL	
Compound 4	Vitamin C	6 g	Dissolved and then add to above mixture
	Multi-vitamins	16 g	
	Water	200 mL	
Compound 5	Formaldehyde	4 mL	Add to above mixture
	Acetic acid	8 mL	

Table S2. Moth feed of *Helicoverpa armigera*

Ingredient	amount
Agar powder	3 g
Casein	8 g
Sucrose	25 g
Water	500 mL
